# The prevalence of mental disorders and patterns of comorbidity within a large sample of mentally ill prisoners: A network analysis

**DOI:** 10.1192/j.eurpsy.2020.63

**Published:** 2020-06-11

**Authors:** Nora van Buitenen, Chantal J.W. van den Berg, Jesse Meijers, Joke M. Harte

**Affiliations:** 1 Judicial Complex Zaanstad, Westzaan, The Netherlands; 2 Department of Criminal Law and Criminology, Vrije Universiteit Amsterdam, Amsterdam, The Netherlands

**Keywords:** Clinical phenotypes, comorbidity, forensic psychiatry, network analysis, psychopathology

## Abstract

**Background.:**

Comorbidity has profound implications in both the clinical field and research, yet little is known about the prevalence and structure of comorbid mental disorders. This article aims not only to present data on the prevalence of mental disorders and comorbidity, but also to explore relationships between comorbid mental disorders by using a network approach.

**Methods.:**

Data used in this cross-sectional study are part of a prospective cohort study within penitentiary psychiatric centers (PPCs) in the Netherlands. It includes DSM diagnoses of 5,257 unique male patients incarcerated in one of the PPC's. Prevalence rates of mental disorders and comorbidity were calculated, the network of comorbid DSM diagnoses was constructed using regression coefficients.

**Results.:**

Schizophrenia spectrum and substance-related disorders were most prevalent within this sample (56.7 and 43.1%, respectively), and over half of all patients were diagnosed with a comorbid disorder (56.9%). Four distinctive groups of disorders emerged from the network analysis of DSM diagnoses**: substance use, impulsivity, poor social skills, and disruptive behaviors. Psychotic disorders were considered as a separate group as it was unconnected to other disorders.

**Conclusions.:**

Comorbid mental disorders can be described, at least in part, as connected networks. Underlying attributes as well as direct influences of mental disorders on one another seem to be affecting the presence of comorbidity. Results could contribute to the understanding of a possible causal relation between psychopathology and criminal behavior and the development of treatment programs targeting groups of disorders.

## Introduction

Comorbidity, defined as the co-occurrence of two or more psychiatric disorders [1], is a widespread phenomenon within the clinical practice of psychiatry and psychology. Previous studies indicated high rates of comorbidity in various populations with mental illness. The United States National Comorbidity Survey found that 56% of adults with a Diagnostic and Statistical Manual of Mental Disorders (DSM) diagnosis at some point in their lives had also been diagnosed with another mental disorder [2].

Clinical effects of comorbidity are profound. Comorbidity has negative effects on the progression of a disorder, the severity of symptoms [3–6], and treatment outcome [7, 8], and is associated with more antisocial behavior [3,9]. Patients with comorbid mental disorders experience more functional disability [4,10], less social competence [6,11], and higher public service utilization [6,12]. Comorbidity also causes multiple complications for research. For example, restricting a study to “pure cases” (i.e., subjects with only one mental disorder) limits the generalizability and ability to detect correlates of mental disorders. Furthermore, studying samples with a target disorder, and disregarding the presence of comorbid disorders, compromises the interpretation of all results as the confounding influence of comorbid mental disorders is not considered [13].

Although complications associated with comorbidity are evident, current psychiatric classification systems such as the DSM or International Classification of Diseases still describe mental disorders as discrete conditions with little respect to the overlap in symptoms of various mental disorders. Different approaches to understanding and classifying psychopathology have been proposed. Among them is the common cause hypothesis, in which comorbidity is explained as the effect of a latent attribute [14]. Studies using latent class analysis have yielded distinctive profiles of comorbidity [13,15]. However, the common cause hypothesis does not allow for a direct relationship between the observable indicators, and it is questionable whether this is plausible [14]. Consider, for example, the relationship between insomnia and psychiatric disorders, that is, insomnia can lead to depression, anxiety, or even psychosis. It seems that the relationship between mental disorders could also emerge as a result of direct influences they impose on each other.

To define and analyze relationships between various mental disorders, without postulating the existence of a common cause, Cramer et al. [14] proposed a network approach to comorbidity. Based on network models of depression and anxiety, they argue that it “generates realistic hypotheses about pathways to comorbidity, overlapping symptoms, and diagnostic boundaries, that are not naturally accommodated by latent variable models” (p. 33).

Currently, around 585,000 individuals are incarcerated in European prisons, of which 10,000 are incarcerated in the Netherlands [16]. The Netherlands has one of the lowest incarceration rates in Europe, with 50 detainees per 100,000 inhabitants [16,17]. This is a much lower rate than the European average of 118.5 [16] and comparable to, for example, Sweden and Finland [17]. Around 60% of the total Dutch prison population has been diagnosed with a mental disorder [17]. As noted before, comorbidity is associated with more antisocial behavior, and it has been suggested that psychiatric patients with multiple disorders are at a higher risk of being incarcerated [18,19]. Prevalence rates of comorbidity as high as 90% in prisons seem to support this notion. Combinations of schizophrenia and bipolar disorder with antisocial personality and substance use disorders (SUD) are common within this population [3], although comorbidity research has been limited in this population [20].

The aim of this study is to present data on the prevalence of mental disorders and comorbidity in a large sample of mentally ill prisoners (*n* = 5,257). To take into consideration comorbidity, we also model a network of DSM diagnoses and their relations to each other and explore the possibility of underlying attributes and direct relations between mental disorders. This study will provide a better understanding of serious mental illness and the structure of comorbidity within a forensic population.

## Materials and Methods

### Penitentiary psychiatric centers and the National Database PPC

The data used in this study were collected in the four penitentiary psychiatric centers (PPC) in the Netherlands. PPCs are facilities within the Dutch criminal justice system equipped to house detainees who are incapable of functioning within a regular prison regime due to their mental state and need specialized psychiatric care [21].

Since May 1, 2013, the PPCs are required to systematically gather information on all patients admitted to the PPC, resulting in the National Database PPC. The database contains diagnostic information, demographic patient characteristics, and criminal records, as well as information on clinical symptoms. The data are used for policymaking and evaluation of the effectiveness of treatment offered to patients. This study uses data collected within the clinical practice and comprises secondary analyses of these data for scientific research.

### Ethical considerations

The secondary usage of anonymized data for scientific research, as presented in this paper, was authorized by the Dutch Ministry of Justice and Security. Additionally, the Ethics Committee of the Department of Law and Criminology, Vrije Universiteit Amsterdam, approved this study.

### Participants

This study includes data of all 9,057 patients detained in the four PPCs in the Netherlands between May 1, 2013, and June 2, 2019. In case of multiple admittances of the same person, which is not unusual in this population, data gathered during the most recent admittance was included in the study. Removing data of 3,271 previous admissions resulted in a sample of 5,786 unique subjects. Given possible gender differences in diagnosis and comorbidity [22], only male patients were included in this study, resulting in a final sample size of 5,257 patients. Offenses committed by this sample are omnifarious, ranging from felony drug charges to homicide.

### DSM diagnosis

Upon admittance to the PPC, both a psychiatrist and a psychologist conduct an independent primary interview with the patient. The final DSM diagnosis is the result of a consensus diagnosis between these two professionals. As of January 1, 2017, the DSM-IV is replaced by the DSM-5 to classify the presence of a mental disorder. For a detailed description on the merging of DSM-IV and DSM-5 diagnoses, see Appendix A.

All diagnoses were used to establish the prevalence of mental disorders, including a “deferred diagnosis” either on axis I, axis II, or both. Diagnoses were deferred when it was not possible to conclude a diagnosis, whereas the presence of a mental illness seems likely. To make no assumptions about the presence of a mental disorder [21], we excluded deferred diagnoses from calculations on the prevalence of comorbidity, resulting in a sample size of *n* = 5,133 of comorbidity assessment.

The numerous DSM diagnoses had to be categorized for the network analysis, retaining both clinical relevance and an adequate sample size. See Appendix A for a detailed account of this categorization. Categories with an insufficient sample size (*n* < 20) that could not be merged without compromising clinical relevance were excluded from the network analysis. Some patients (*n* = 68) were exclusively diagnosed with disorders belonging to these small categories, resulting in exclusion. Data of 5,065 patients were included in the network analysis.

### Statistical analyses

Descriptive statistics and prevalence rates were calculated using SPSS (v24; IBM, Armonk, NY). Network analyses were conducted in R.

Because of the binary nature of the data, the *IsingFit* package [23] was used to estimate the network parameters. Based on logistic regressions, the best-fitting function was selected using the extended Bayesian information criterion (EBIC), which has shown to estimate the most relevant features of a network successfully. To ensure the sparsity of the network and to cope with the problem of multicollinearity and multiple testing, all regression coefficients are penalized using *eLasso*, resulting in more conservative network structures. The hyperparameter, which determines the strength of this extra penalty, was set to 0.25 [24,25].

The resulting matrix was plotted using *qGraph* [26]. The network nodes represent categories of DSM diagnoses. The edges represent the reciprocal relations (AND rule) between the DSM diagnoses (i.e., the presence of comorbidity between diagnoses). Only positive estimates were included in the model to clearly describe patterns of comorbidity between diagnoses, as these relations are indicated by the positive edges. In the visualization of the network, the Fruchterman–Reingold algorithm was used, which places strongly connected nodes close to each other [25,27].

Possible clinical phenotypes, indicated by groups of nodes clustering together, were investigated using the Walktrap algorithm, which has shown to perform well in psychological networks [28].

## Results

### Prevalence of mental illness and comorbidity

Prevalence rates of mental disorders are displayed in Table 1. Prevalence rates of comorbidity rates found in this sample are displayed in Table 2. In total, 56.9% of the patients had one or more comorbid diagnoses.

**Table 1.** Prevalence rates of mental disorders in a sample of mentally ill offenders.

Diagnosis	*N*	%
Deferred diagnosis axis I	202	3.8
Deferred diagnosis axis II	2,014	38.3
Neurodevelopmental disorders	822	15.6
Schizophrenia spectrum and other psychotic disorders	2,979	56.7
Bipolar and related disorders	231	4.4
Depressive disorders	264	5.0
Anxiety disorders	65	1.2
Obsessive–compulsive and related disorders	19	0.4
Trauma- and stressor-related disorders	442	8.4
Dissociative disorders	6	0.1
Somatic symptom and related disorders	20	0.4
Feeding and eating disorders	4	0.1
Elimination disorders	1	0.0
Sleep–wake disorders	5	0.1
Gender dysphoria	6	0.1
Disruptive, impulse-control, and conduct disorders	141	2.7
Substance-related and addictive disorders	2,265	43.1
Neurocognitive disorders	68	1.3
Paraphilic disorders	61	1.2
Other mental disorders	124	2.4
Movement disorders and other side effects	2	0.0
Cluster A (odd or eccentric disorders)		
Cluster A unspecified	7	0.1
Paranoid personality disorder	10	0.2
Schizotypal personality disorder	10	0.2
Schizoid personality disorder	9	0.2
Cluster B (dramatic, emotional, or erratic disorders)		
Cluster B unspecified	131	2.5
Antisocial personality disorder	358	6.8
Borderline personality disorder	169	3.2
Narcissistic personality disorder	48	0.9
Histrionic personality disorder	4	0.1
Cluster C (anxious or fearful disorders)		
Cluster C unspecified	15	0.3
Dependent personality disorder	6	0.1
Avoidant personality disorder	10	0.2
Obsessive–compulsive personality disorder	2	0.0
Other specified personality disorder	51	1.0
Unspecified specified personality disorder	618	11.8

*N* = 5,257.

**Table 2.** Prevalence rates of comorbid mental disorders in a sample of mentally ill offenders.

Number of diagnosis	*n*	%
1	2,214	43.1
2	1,538	30.0
3	840	16.4
4	391	7.6
5	112	2.2
6	27	0.5
7	10	0.2
8	1	0.0

Prevalence rates of comorbidity when deferred diagnoses are excluded (*n* = 5,133).

### Network of DSM diagnosis

**Figure 1.**
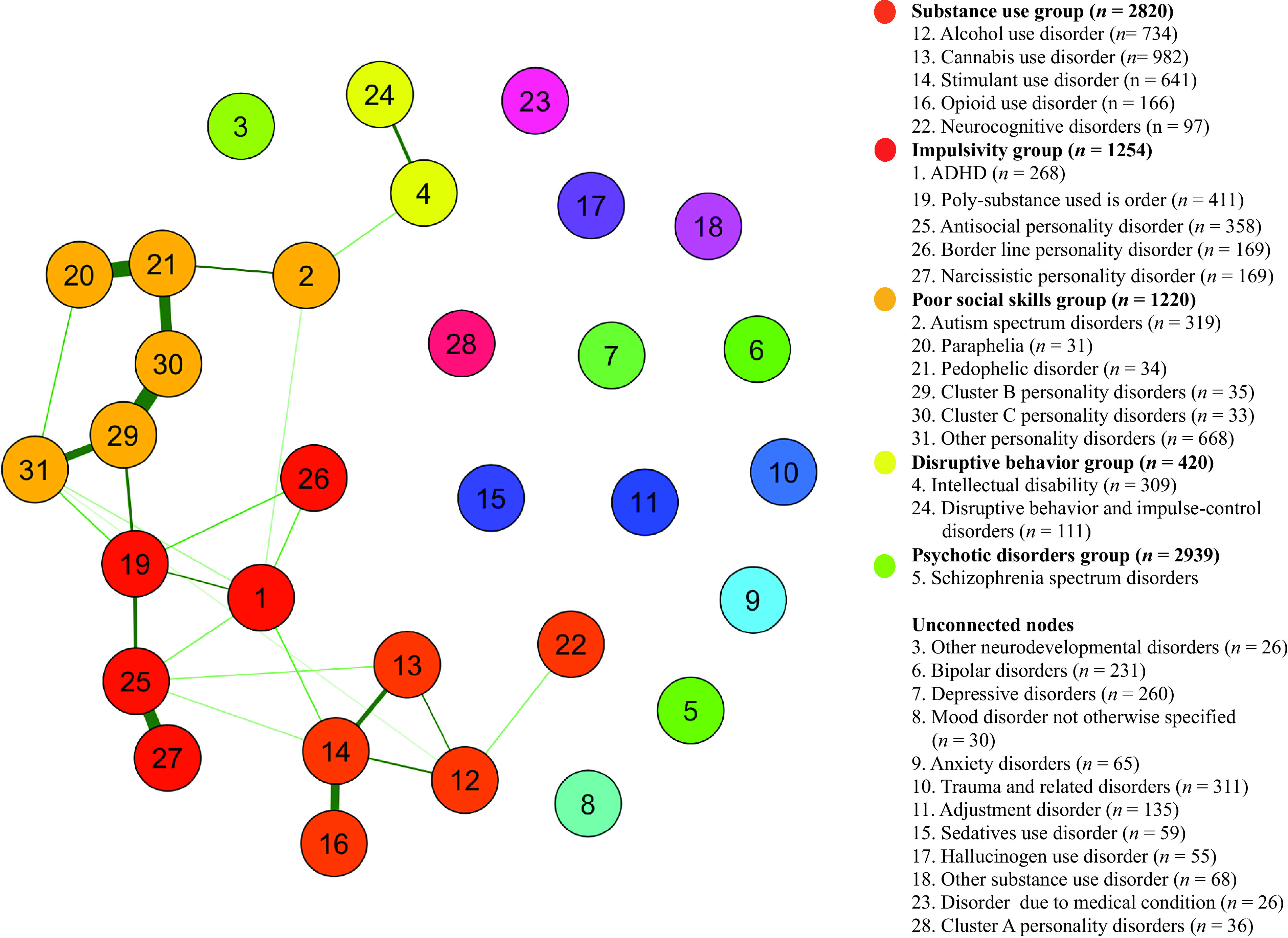
Network model of Diagnostic and Statistical Manual of Mental Disorders diagnoses. Abbreviation: ADHD, attention-deficit/hyperactivity disorder. *n* = 5,060.

The final network model (see Figure 1) included DSM data of 5,065 patients in 31 categories of mental disorders. Thick edges represent strong correlations. Node names and sample sizes are listed in the legend. The network shows 18 connected categories and reveals four communities of mental disorders (connected groups of diagnostic categories).

### Clusters of comorbidity

The largest community is formed by alcohol use, cannabis use, opioid use, and stimulant use disorders, and neurocognitive disorders. It accounts for 26.9% of all observations of DSM diagnoses within the network. This *substance use group* represents excessive substance use and its probable neurocognitive consequences. Both cannabis use disorders and stimulant use disorders are connected to antisocial personality disorder (ASPD). Furthermore, stimulant use disorder is connected to the node that represents attention-deficit/hyperactivity disorder (ADHD), forming a bridge to the second group.

The second group is defined as the *impulsivity group*, accounting for 12.9% of all observations. It includes ASPD, narcissistic personality disorder, ADHD, borderline personality disorder, and polysubstance use. Besides high levels of impulsivity, this group includes patients portraying a lifetime pattern of antisocial behaviors. Polysubstance use forms a bridge to the third group by correlating with other and cluster B personality disorders. Another bridge to the third group is formed by the connections between ADHD and both autism spectrum disorders (ASDs) and other personality disorders.

The third group accounts for 12.5% of all observations and includes ASDs, cluster B personality disorders, cluster C personality disorders, other personality disorders, paraphilia, and pedophilia. This group might experience eccentric sexual preferences and could be expected to portray below-average social skills. Therefore, it is deemed the *poor social skills group.* It connects to the *impulsivity group* through several edges, including connections with ADHD, a disorder also often characterized by difficulties in social interactions.

The last connected group is the *disruptive behaviors group* and contains intellectual disabilities and disruptive, impulse-control, and conduct disorders. Intellectual disabilities connect to the *poor social skills group* via ASDs. The *disruptive behaviors group* could be characterized by many behavioral problems, most likely starting from a young age. The group’s connection to ASD seems to support this, as ASDs have an early onset and individuals with these disorders often portray disruptive behaviors. The group constitutes 4.3% of all observations.

Finally, several nodes remain unconnected within the network. Schizophrenia spectrum and other psychotic disorders contain the largest amount of all observations (30.1%) within the network. It is therefore viewed at the level of a community and defined as the *psychotic disorders group.*

The five described diagnostic groups sum up to 86.7% of all observed DSM diagnoses. The remaining nodes that are unrelated to the network represent 13.3%.

## Discussion

The results of this study revealed high levels of comorbidity within a sample of mentally ill prisoners. Taken together, 56.9% of the patients had one or more comorbid diagnoses. These results are in line with the sparse previous research on the prevalence rates of comorbidity within correctional settings [20,22], where it should be noted that these rates strongly vary across countries and specific settings. For example, in an older study in Quebec, Canada, researchers found that 386 of the 495 interviewed male prisoners suffered from a comorbid mental disorder, ranging from 84 to 94% for specific disorders [3]. High rates of comorbid mental disorders were also found in Chilean prisoners, where the highest rate of comorbid disorders (92%) was found for major depressive disorders [29]. Last, in their systematic review, Fazel and Seewald found ranges of 20–44% for the co-occurrence of substance misuse in prisoners with mental illness [20]. Our study presents a novel approach for modeling DSM diagnoses and comorbidity within this complex sample. Results of the network analysis show four distinctive diagnostic communities and one substantial distinctive unconnected diagnostic category.

The finding that various addictive disorders tend to cluster together in the *substance use group* is not surprising. Studies have demonstrated high rates of comorbid SUDs [30], and its presence increases the risk of having another addictive disorder [31]. The connections between opioid, stimulant, and cannabis disorder are interesting, as these substances have an opposite effect. A possible explanation could be that one tries to attenuate vigorous effects of one drug with another [32]. In addition, the relationship between substance use and criminal behavior is a well-established one [33,34]. Consequently, the prevalence rates of SUD within prison samples are high [35,36], as was found in this study (43.1%).

The *impulsivity group* is described as impulsive and externalizing. Previous research indicates a relationship between ADHD and cluster B personality disorders [37,38] and increased rates of substance abuse in both disorders [38–40]. It has been proposed that the relation between ADHD and substance use is, partially, mediated by the presence of antisocial disorders [38]. In the current network, both ADHD and ASPD are “bridge-connections” to the *substance use group* and are also connected to each other. The connection of ADHD to stimulant use is interesting, as stimulants have been proposed to be used as self-medication by individuals with ADHD [41]. Finally, the strong connection between antisocial and narcissistic personality disorders should be noted. The disorders have shown many similarities and have been questioned as discrete conditions [42]. It is not hard to imagine how an individual belonging to the *impulsivity group* would be at risk of portraying criminal behavior given the described interplay of impulsivity, substance use, and antisocial personality structures.

The *poor social skills group* is defined as a group of below-average social skills and patients portraying eccentric sexual behaviors. The group shows the strongest correlations between the diagnostic categories it includes, which may be explained by the overlap in diagnostic criteria for personality disorders in the DSM-IV [43]. Of much interest is the connection between the cluster C personality disorders and the presence of a pedophilic disorder. These personality disorders seem to describe a shy and socially isolated sex offender, which may limit them in establishing intimate relationships [44], perhaps resulting in gravitation toward younger children. Also interesting is the connection between sexual disorders and ASDs. Unfortunately, not much is known about the possible relation between paraphilia, including pedophilia, and ASDs, but some research found some deviant sexual behaviors in young males with an ASD [45]. It seems that there are possible connections between sexual offending and mental disorders that are relevant to explore. Besides the obvious risk of criminal offending related to certain sexual dysfunctions, limited social skills have also been related to criminal offending [46]. The current results underline the possible importance of improving the social skills of the offenders within this group. It should be noted that this group also includes a small sample of cluster B personality disorders, a condition not usually described by substandard social skills. Given its position close to the *impulsivity group*, it is a bridge-connection to this group.

The *disruptive behavior group* is characterized by the presence of many behavioral problems, including aggressive and destructive behaviors. Previous research has indicated that individuals with an intellectual disability showed increased prevalence rates of ASDs and conduct disorders [47], and both connections are also found in the current model. Aggression and criminal behavior are associated with both intellectual disabilities in adults [48] and history of conduct disorder in patients with a severe mental illness [49]. When considering direct relationships between mental disorders, one could contemplate whether disruptive, impulse-control, and conduct disorders are, in some way, an expression of intellectual disabilities.

Finally, the most prevalent is the *psychotic disorder group* (56.7%). High rates of psychotic disorders in prison samples have been found by previous studies [20,21] and have been related to criminal [50–52] and often violent behavior [50,53–55]. It should be noted that the *psychotic disorder group* is not connected to other diagnostic categories within the network model, even though a comorbid SUD is very common in psychotic disorders [22,52,56] and is seen as a major risk factor of violent behavior in psychotic individuals [57].

The five groups indicate that comorbidity is not only influenced by latent constructs, as is proposed by the common cause hypothesis. Although latent constructs could be derived from the findings of this study, it also shows how comorbidity can be a result of direct effects that various mental disorders impose on one another. The described groups provide a first step in the development of clinical phenotypes of mentally ill offenders based on their comorbid psychopathology.

### Limitations

It should be noted that possible associations and patterns of comorbidity exist that are not represented in this sample or network. For example, a relation between the *psychotic disorder group* and the *substance use group* was expected. One explanation for its absence could be that clinical professionals are inclined to underdiagnose comorbid mental illness next to a psychotic disorder, as the presence of a florid psychosis may interfere with the diagnostic process. The high prevalence of deferred diagnoses (38.3%) seems to support this notion. Similar explanations could be possible for other absent connections.

The data used in this study are part of a large and unique cohort of mentally ill prisoners and are an accurate representation of the clinical practice. The data could, however, possibly obscure certain expected connections and prevalence rates. The absence of structurally gathered data for research purposes, for example, by using the Structured Clinical Interview for DSM-IV, is a significant limitation in this study.

An alternative explanation for the absence of expected relations between mental disorders is the conservative method of modeling. The use of *eLasso* highly penalizes connections in the network, and only reciprocal relations were included. Networks of the data modeled with more lenient methods indicated more connections, among them a connection between the *psychotic disorder group* and *substance use* group. However, a conservative method of modeling was deemed more fitting, given the exploratory nature of this study.

### Future research

This study is a first attempt at modeling the structure of the comorbidity of DSM diagnoses of this complex sample by using a network analysis approach. Future research should focus on replication, optimization, and expansion of possible clinical phenotypes based on psychopathology, preferably using data gathered for research purposes. The development of clinical phenotypes is of great importance for the prevention of criminal behavior by mentally ill offenders as they could provide information about risk factors of criminal behavior associated with mental illness. This information could provide markers for early identification of individuals at risk of portraying criminal behavior as a consequence of the structure of their (comorbid) mental disorders.

Comorbidity has profound clinical effects. Treatment of clusters of psychopathology, taking into account interactions between comorbid disorders, may yield more results in improving mental health and reducing recidivism rates of mentally ill offenders. More knowledge on the structure of comorbidity is needed to develop treatment programs effectively targeting multiple disorders, or clinical phenotypes. This would not only benefit the forensic field, but also the field of psychiatry as a whole.

## Data Availability

The authors received permission from the Dutch Ministry of Justice and Security to access the data used in this study. However, they are unable to share the data as they are not the data custodians.

## Appendix A

As of January 1, 2017, the Diagnostic and Statistical Manual of Mental Disorders−Fifth Edition (DSM-5) is used to assess the presence of a mental disorder in the Netherlands. Before this date, the DSM-IV was used for this purpose. Due to this change in classifications systems, the data used in this study consist of both DSM-IV and DSM-5 scores. For all subjects included in this study, International Classification of Diseases−Ninth Revision (ICD-9) codes were available. The ICD-9 codes were linked to specific mental disorders and their description in either DSM-IV, DSM-5, or both if the code appeared in both versions.

The linked codes were examined by trained professionals, both in the field of forensic psychiatry and forensic psychology, and recoded in overarching categories ensuring both clinical relevance of the categories and a sufficient sample size within them for further analysis. Most ICD-9 codes were present in both DSM-IV and DSM-5 and had a similar description in both DSM versions. If the ICD-9 code referenced to fundamentally different descriptions of mental disorders within the versions of the DSM, we indicated to which version of the DSM the ICD-9 code included in the category refers. Thus, resulting in the following 31 categories:

### Attention-deficit/hyperactivity disorder

314.00 ADHD, predominantly inattentive presentation.

314.01 ADHD, combined presentation.

ADHD, predominantly hyperactive/impulsive presentation.

Other specified ADHD.

Unspecified ADHD.

314.90 ADHD not otherwise specified (NOS).

### Autism spectrum disorders

299.00 Autistic disorder.

Autism spectrum disorder.

299.80 Rett’s disorder.

Asperger’s disorder.

Pervasive developmental disorder NOS.

### Other neurodevelopmental disorders

307.00 Adult-onset fluency disorder.

Stuttering.

307.20 Unspecified tic disorder.

Other specified tic disorder.

Tic disorder NOS.

307.22 Persistent (chronic) motor or vocal tic disorder.

307.23 Tourette’s disorder.

307.90 Unspecified communication disorder.

Communication disorder NOS.

315.00 Specific learning disorder, with impairment in reading.

Learning disorder.

315.10 Specific learning disorder, with impairment in mathematics.

Mathematics disorder.

315.39 Language disorder.

Social (pragmatic) communication disorder.

Speech sound disorder.

Phonological disorder.

315.80 Other specified neurodevelopmental disorder.

Global developmental delay.

315.90 Unspecified neurodevelopmental disorder.

Stuttering.

Learning disorder NOS.

### Intellectual disability

317.00 Intellectual disability (intellectual developmental disorder), mild.

Mild mental retardation.

318.00 Intellectual disability (intellectual developmental disorder), moderate.

Moderate mental retardation.

318.10 Intellectual disability (intellectual developmental disorder), severe.

Severe mental retardation.

319.00 Intellectual disability (intellectual developmental disorder).

Mental retardation; severity unspecified.

### Schizophrenia spectrum and other psychotic disorders

291.30 Alcohol-induced psychotic disorder, with hallucinations.

291.90 Alcohol-induced psychotic disorder.

292.11 Amphetamine-induced psychotic disorder, with delusions.

Cannabis-induced psychotic disorder, with delusions.

Cocaine-induced psychotic disorder, with delusions.

Hallucinogen-induced psychotic disorder, with delusions.

Inhalant-induced psychotic disorder, with delusions.

Opioid-induced psychotic disorder, with delusions.

Phencyclidine-induced psychotic disorder, with delusions.

Sedative-, hypnotic-, or anxiolytic-induced psychotic disorder, with delusions.

Other substance-induced psychotic disorder, with delusions.

292.12 Amphetamine-induced psychotic disorder, with hallucinations.

Cannabis-induced psychotic disorder, with hallucinations.

Cocaine-induced psychotic disorder, with hallucinations.

Hallucinogen-induced psychotic disorder, with hallucinations.

Inhalant-induced psychotic disorder, with hallucinations.

Opioid-induced psychotic disorder, with hallucinations.

Phencyclidine-induced psychotic disorder, with hallucinations.

Sedative-, hypnotic-, or anxiolytic-induced psychotic disorder, with hallucinations.

Other substance-induced psychotic disorder, with hallucinations.

295.10 Schizophrenia disorganized type.

295.20 Schizophrenia catatonic type.

295.30 Schizophrenia paranoid type.

295.40 Schizophreniform disorder.

295.60 Schizophrenia residual type.

295.70 Schizoaffective disorder, bipolar type.

Schizoaffective disorder, depressive type.

295.90 Schizophrenia.

Schizophrenia undifferentiated type.

297.10 Delusional disorder.

298.80 Brief psychotic disorder.

Other specified schizophrenia spectrum and other psychotic disorder.

298.90 Unspecified schizophrenia spectrum and other psychotic disorder.

Psychotic disorder NOS.

### Bipolar disorder

296.00 Bipolar I disorder, single manic episode unspecified.

296.01 Bipolar I disorder, single manic episode mild.

296.02 Bipolar I disorder, single manic episode, moderate.

296.04 Bipolar I disorder, single manic episode, severe with psychotic features.

296.06 Bipolar I disorder, single manic episode, in full remission.

296.40 Bipolar I disorder, current or most recent episode hypomanic.

Bipolar I disorder, current or most recent episode hypomanic, unspecified.

Bipolar I disorder, current or most recent episode manic, unspecified.

296.41 Bipolar I disorder, current or most recent episode manic, mild.

296.42 Bipolar I disorder, current or most recent episode manic, moderate.

296.43 Bipolar I disorder, current or most recent episode manic, severe.

Bipolar I disorder, current or most recent episode manic, severe without psychotic features.

296.44 Bipolar I disorder, current or most recent episode manic, with psychotic features.

296.45 Bipolar I disorder, current or most recent episode hypomanic, in partial remission.

Bipolar I disorder, current or most recent episode manic, in partial remission.

296.46 Bipolar I disorder, current or most recent episode hypomanic, in full remission.

Bipolar I disorder, current or most recent episode manic, in full remission.

296.50 Bipolar I disorder, current or most recent episode depressed, unspecified.

296.52 Bipolar I disorder, current or most recent episode depressed, moderate.

296.54 Bipolar I disorder, current or most recent episode depressed, with psychotic features.

296.60 Bipolar I disorder, most recent episode mixed unspecified.

296.64 Bipolar I disorder, most recent episode mixed, severe with psychotic features.

296.70 Bipolar I disorder, current or most recent episode unspecified.

296.80 Unspecified bipolar and related disorder.

Bipolar disorder NOS.

296.89 Bipolar II disorder.

Other specified bipolar and related disorder.

### Depressive disorders

296.20 Major depressive disorder, single episode, unspecified.

296.21 Major depressive disorder, single episode, mild.

296.22 Major depressive disorder, single episode, moderate.

296.23 Major depressive disorder, single episode, severe.

Major depressive disorder, single episode, severe, without psychotic features.

296.24 Major depressive disorder, single episode, with psychotic features.

296.25 Major depressive disorder, single episode, in partial remission.

296.26 Major depressive disorder, single episode, in full remission.

296.30 Major depressive disorder, recurrent episode, unspecified.

296.31 Major depressive disorder, recurrent episode, mild.

296.32 Major depressive disorder, recurrent episode, moderate.

296.33 Major depressive disorder, recurrent episode, severe.

296.34 Major depressive disorder, recurrent episode, with psychotic features.

296.35 Major depressive disorder, recurrent episode, in partial remission.

296.36 Major depressive disorder, recurrent episode, in full remission.

300.40 Persistent depressive disorder (dysthymia).

Dysthymic disorder.

311.00 Unspecified depressive disorder.

Other specified depressive disorder.

Depressive disorder NOS.

### Mood disorder NOS

296.90 *Only if diagnosed under DSM-IV*.

### Anxiety disorders

300.00 Unspecified anxiety disorder.

Anxiety disorder.

300.01 Panic disorder.

Panic disorder without agoraphobia.

300.02 Generalized anxiety disorder.

300.21 Panic disorder with agoraphobia.

300.22 Agoraphobia.

Agoraphobia without history of panic disorder.

300.23 Social anxiety disorder (social phobia).

300.29 Specific phobia, animal.

Specific phobia, blood–injection–injury.

Specific phobia, natural environment.

Specific phobia, other.

Specific phobia, situational.

Specific phobia.

### Trauma and stressor-related disorders

308.30 Acute stress disorder.

309.81 Posttraumatic stress disorder.

309.89 Other specified trauma- and stressor-related disorder.

309.90 Unspecified trauma- and stressor-related disorder.

Adjustment disorder, unspecified.

313.89 Disinhibited social engagement disorder.

Reactive attachment disorder.

### Adjustment disorder

309.00 Adjustment disorder, with depressed mood.

309.00 Adjustment disorder, with mixed disturbance of emotions and conduct.

309.24 Adjustment disorder, with anxiety.

309.28 Adjustment disorder, with mixed anxiety and depressed mood.

309.30 Adjustment disorder, with disturbance of conduct.

309.40 Adjustment disorder with mixed disturbance of emotions and conduct.

### Alcohol use disorder

291.89 Alcohol-induced anxiety disorder.

Alcohol-induced bipolar and related disorder.

Alcohol-induced depressive disorder.

Alcohol-induced mild neurocognitive disorder.

Alcohol-induced sexual dysfunction.

Alcohol-induced sleep disorder.

Alcohol-induced mood disorder.

303.00 Alcohol intoxication.

303.90 Alcohol use disorder, moderate.

Alcohol use disorder, severe.

Alcohol-related disorders, dependence.

305.00 Alcohol use disorder, mild.

Alcohol abuse.

### Cannabis use disorder

304.30 Cannabis use disorder, moderate.

Cannabis use disorder, severe.

Cannabis-related disorders, dependence.

305.20 Cannabis use disorder, mild.

Cannabis use disorder.

Cannabis abuse.

### Stimulant use disorder

304.20 Cocaine use disorder, moderate/severe.

Cocaine dependence.

304.40 Other or unspecified stimulant use disorder, moderate.

Other or unspecified stimulant use disorder, severe.

Amphetamine-type substance use disorder, moderate.

Amphetamine-type substance use disorder, severe.

Amphetamine-type related disorders, dependence.

305.60 Cocaine use disorder, mild.

Cocaine abuse.

305.70 Other or unspecified stimulant use disorder, mild.

Amphetamine-type substance use disorder, mild.

Amphetamine abuse.

### Sedatives use disorder

304.10 Sedative, hypnotic, or anxiolytic-use disorder, moderate.

Sedative, hypnotic, or anxiolytic use disorder, severe.

Sedative-, hypnotic-, or anxiolytic-related disorders, dependence.

305.40 Sedative, hypnotic, or anxiolytic use disorder, mild.

Sedative, hypnotic, or anxiolytic abuse.

### Opioid use disorder

304.00 Opioid use disorder, moderate/severe.

Opioid dependence.

305.5 Opioid use disorder, mild.

Opioid abuse.

### Hallucinogen use disorder

304.50 Other hallucinogen use disorder, moderate/severe.

Hallucinogen dependence.

304.60 Inhalant use disorder, moderate/severe.

Phencyclidine use disorder, moderate/severe.

Inhalant dependence.

Phencyclidine dependence.

305.30 Other hallucinogen use disorder, mild.

Hallucinogen abuse.

305.90 Inhalant use disorder, mild.

Other (or unknown) substance use disorder, mild.

Phencyclidine use disorder, mild.

### Other substance use disorder

304.9 Other (or unknown) substance use disorder, moderate.

Other (or unknown) substance use disorder, severe.

Other (or unknown) substance-related disorder, dependence.

### Polysubstance dependence

304.8 Polysubstance dependence.

### Paraphilia

302.82 Voyeuristic disorder.

293.83 Sexual masochism disorder.

302.84 Sexual sadism disorder.

302.89 Frotteuristic disorder.

Other specified paraphilic disorder.

302.90 Unspecified paraphilic disorder.

Paraphilia NOS.

### Pedophilic disorder

302.20 Pedophilic disorder.

Pedophilia.

### Neurocognitive disorders

290.40 Vascular neurocognitive disorder, probable, with/without behavioral disturbance.

Vascular dementia uncomplicated with/without behavioral disturbance.

291.10 Alcohol-induced major neurocognitive disorder, amnestic confabulatory type.

Alcohol-induced persisting amnestic disorder.

291.20 Alcohol-induced major neurocognitive disorder, nonamnestic confabulatory type.

Alcohol-induced persisting dementia.

294.10 Major frontotemporal neurocognitive disorder, probable, without behavioral disturbance.

Major neurocognitive disorder due to Alzheimer’s disease, probable, without behavioral disturbance.

Major neurocognitive disorder due to another medical condition, without behavioral disturbance.

Major neurocognitive disorder due to human immunodeficiency virus (HIV) infection, without behavioral disturbance.

Major neurocognitive disorder due to Huntington’s disease, without behavioral disturbance.

Major neurocognitive disorder due to multiple etiologies, without behavioral disturbance.

Major neurocognitive disorder due to Parkinson’s disease, probable, without behavioral disturbance.

Major neurocognitive disorder due to prion disease, without behavioral disturbance.

Diffuse traumatic brain injury with loss of consciousness of unspecified duration.

Major neurocognitive disorder due to traumatic brain injury, without behavioral disturbance.

Major neurocognitive disorder with Lewy bodies, probable, without behavioral disturbance.

Dementia of the Alzheimer’s type, without behavioral disturbance.

Dementia due to HIV disease, without behavioral disturbance.

Dementia due to head trauma, without behavioral disturbance.

Dementia due to Parkinson’s disease, without behavioral disturbance.

Dementia due to Huntington’s disease, without behavioral disturbance.

Dementia due to Pick’s disease, without behavioral disturbance.

Dementia due to Creutzfeldt–Jakob disease, without behavioral disturbance.

Dementia due to other general medical condition, without behavioral disturbance.

294.11 Major frontotemporal neurocognitive disorder, probable, with behavioral disturbance.

Major neurocognitive disorder due to Alzheimer’s disease, probable, with behavioral disturbance.

Major neurocognitive disorder due to another medical condition, with behavioral disturbance.

Major neurocognitive disorder due to HIV infection, with behavioral disturbance.

Major neurocognitive disorder due to Huntington’s disease, with behavioral disturbance.

Major neurocognitive disorder due to multiple etiologies, with behavioral disturbance.

Major neurocognitive disorder due to Parkinson’s disease, probable, with behavioral disturbance.

Major neurocognitive disorder due to prior disease, with behavioral disturbance.

Major neurocognitive disorder due to traumatic brain injury, with behavioral disturbance.

Major neurocognitive disorder with Lewy bodies, probable, with behavioral disturbance.

Dementia of the Alzheimer’s type, with behavioral disturbance (early/late).

Dementia due to HIV disease, with behavioral disturbance.

Dementia due to head trauma, with behavioral disturbance.

Dementia due to Parkinson’s disease, with behavioral disturbance.

Dementia due to Huntington’s disease, with behavioral disturbance.

Dementia due to Pick’s disease, with behavioral disturbance.

Dementia due to Creutzfeldt–Jakob disease, with behavioral disturbance.

Dementia due to other general medical condition, with behavioral disturbance.

294.90 *Only if diagnosed under DSM-IV*: cognitive disorder NOS.

331.83 Mild frontotemporal neurocognitive disorder.

Mild neurocognitive disorder due to Alzheimer’s disease.

Mild neurocognitive disorder due to another medical condition.

Mild neurocognitive disorder due to HIV infection.

Mild neurocognitive disorder due to Huntington’s disease.

Mild neurocognitive disorder due to multiple etiologies.

Mild neurocognitive disorder due to Parkinson’s disease.

Mild neurocognitive disorder due to prion disease.

Mild neurocognitive disorder due to traumatic brain injury.

Mild neurocognitive disorder with Lewy bodies.

Mild vascular neurocognitive disorder.

799.59 Unspecified neurocognitive disorder.

### Mental disorder due to another medical condition

293.00 Delirium due to another medical condition.

Delirium due to multiple etiologies.

293.81 Psychotic disorder due to another medical condition, with delusions.

293.90 Mental disorder NOS due to general medical condition.

294.00 Amnestic disorder due to general medical condition.

294.80 Obsessive–compulsive and related disorder due to another medical condition.

Other specified mental disorder due to another medical condition.

294.90 *Only if diagnosed under DSM-5*: unspecified mental disorder due to another medical condition.

310.10 Personality change due to another medical condition.

### Disruptive behavior and impulse-control disorders

312.30 Impulse-control disorder NOS.

312.33 Pyromania.

312.34 Intermittent explosive disorder.

312.81 Conduct disorder, childhood-onset type.

312.82 Conduct disorder, adolescent-onset type.

312.89 Conduct disorder, unspecified onset.

Other specified disruptive, impulse-control, and conduct disorder.

312.90 Unspecified disruptive, impulse-control, and conduct disorder.

Disruptive behavior disorder NOS.

313.81 Oppositional defiant disorder.

### Antisocial personality disorder

301.70.

### Borderline personality disorder

301.83.

### Narcissistic personality disorder

301.81.

### Cluster A personality disorders

301.20 Schizoid personality disorder.

301.22 Schizotypal personality disorder.

301.00 Paranoid personality disorder.

If cluster A is defined, but not the specific personality disorder, data are included in this category.

### Cluster B personality disorders

301.50 Histrionic personality disorder is included in this category because of the very low prevalence of the disorder within this sample.

If cluster B is defined, but not the specific personality disorder, data are included in this category.

### Cluster C personality disorders

301.60 Dependent personality disorder.

301.82 Avoidant personality disorder.

301.40 Obsessive–compulsive personality disorder.

If cluster C is defined, but not the specific personality disorder, data are included in this category.

### Other personality disorders

301.90 Unspecified personality disorder.

Personality disorder NOS.

301.89 Otherwise specified personality disorder.

The following categories were excluded from the network analysis because of a small sample size within the category (*n* < 20).

### Delirium (n = 11)

291.00 Alcohol intoxication delirium.

Alcohol withdrawal delirium.

292.81 Medication-induced delirium.

Opioid intoxication delirium.

Other (or unknown) substance intoxication delirium.

Hallucinogen intoxication delirium.

Phencyclidine intoxication delirium.

Sedative, hypnotic, or anxiolytic intoxication delirium.

Sedative, hypnotic, or anxiolytic withdrawal delirium.

Amphetamine (or other stimulant) intoxication delirium.

Cannabis intoxication delirium.

Cocaine intoxication delirium.

Inhalant intoxication delirium.

780.09 Unspecified delirium.

Other specified delirium.

Delirium NOS.

### Dissociative disorders (n = 6)

300.14 Dissociative identity disorder.

300.15 Other specified dissociative disorder.

Unspecified dissociative disorder.

Dissociative disorder NOS.

### Eating disorders (n = 4)

307.10 Anorexia nervosa.

307.50 Unspecified feeding or eating disorder.

Eating disorder NOS.

### Gender dysphoria (n = 6)

302.60 Gender dysphoria in children.

Other specified gender dysphoria.

Unspecified gender dysphoria.

Gender identity disorder in children.

Gender identity disorder NOS.

302.85 Gender dysphoria in adolescents and adults.

### Sleep disorder (n = 5)

307.42 Primary insomnia.

307.47 Nightmare disorder.

Dyssomnia NOS.

Parasomnia NOS.

780.52 Insomnia disorder.

Other specified insomnia disorder.

Unspecified insomnia disorder.

### Somatic symptom disorders (n = 18)

300.11 Conversion disorder.

300.82 Somatic symptom disorder.

Unspecified somatic symptom and related disorder.

Somatoform disorder NOS.

307.89 Pain disorder associated with both psychological factors and a general medical condition.

### Somatoform disorders (n = 2)

300.7 Body dysmorphic disorder.

Illness anxiety disorder.

Hypochondriasis.

### Obsessive–compulsive disorder (n = 17)

300.3 Obsessive–compulsive disorder.

Other specified obsessive–compulsive and related disorder.

Unspecified obsessive–compulsive and related disorder.

Hoarding disorder.

312.39 Trichotillomania.

### Withdrawal (n = 14)

291.81 Alcohol withdrawal.

292.00 Amphetamine or other stimulant withdrawal.

Caffeine withdrawal.

Cannabis withdrawal.

Cocaine withdrawal.

Opioid withdrawal.

Opioid withdrawal delirium.

Other (or unknown) substance withdrawal.

Other (or unknown) substance withdrawal delirium.

Sedative, hypnotic, or anxiolytic withdrawal.

Sedative, hypnotic, or anxiolytic withdrawal delirium.

Tobacco withdrawal.

### Gambling disorder (n = 17)

312.31 Gambling disorder.

### Substance-induced mood disorder (n = 4)

292.84 Amphetamine (or other stimulant) induced bipolar and related disorder.

Amphetamine (or other stimulant) induced depressive disorder.

Cocaine-induced bipolar and related disorder.

Cocaine-induced depressive disorder.

Inhalant-induced depressive disorder.

Opioid-induced depressive disorder.

Other (or unknown) substance-induced bipolar and related disorder.

Other (or unknown) substance-induced depressive disorder.

Other hallucinogen-induced bipolar and related disorder.

Other hallucinogen-induced depressive disorder.

Phencyclidine-induced bipolar and related disorder.

Phencyclidine-induced depressive disorder.

Sedative, hypnotic, or anxiolytic-induced bipolar and related disorder.

Sedative, hypnotic, or anxiolytic-induced depressive disorder.

Amphetamine-induced mood disorder.

Cocaine-induced mood disorder.

Hallucinogen-induced mood disorder.

Inhalant-induced mood disorder.

Opioid-induced mood disorder.

Phencyclidine-induced mood disorder.

Sedative-, hypnotic-, or anxiolytic-induced mood disorder.

Other substance-induced mood disorder.

### Movement disorder (n = 5)

293.89 Unspecified catatonia.

Catatonia associated with another mental disorder.

Catatonic disorder due to another medical condition.

333.72 Medication-induced acute dystonia.

Tardive dystonia.

333.85 Tardive dyskinesia.

### Factitious disorder (n = 2)

300.19

### Unspecified sexual dysfunction (n = 0)

302.7 Unspecified sexual dysfunction.

Sexual dysfunction NOS.

### Elimination disorders (n = 1)

307.6 Enuresis

307.7 Encopresis.

### Tobacco use disorders (n = 17)

305.10 Tobacco use disorder.

Nicotine dependence.

Finally, three categories of mental disorders were excluded for other reasons than small sample size. ICD-9 codes 292.89 and 292.90 reference to a variety of substance-induced disorders. It was not possible to trace back to what specific description of mental disorders the codes had referenced to when diagnosed. Because of the diffuse character of the resulting category, it was excluded from further analysis. The category “Unspecified mental disorders” was excluded because of its indistinct nature; it provides no information on specific symptoms or behaviors related to the category. Keeping in mind the aim of the current study, to provide possible clinical phenotypes of psychopathology within this population, the variable would not have contributed to this goal.

### Substance-induced other disorders (n = 76)

292.89 Inhalant-induced mild neurocognitive disorder.

Opioid intoxication.

Opioid-induced anxiety disorder.

Opioid-induced sexual dysfunction.

Other (or unknown) substance intoxication.

Other (or unknown) substance-induced anxiety disorder.

Other (or unknown) substance-induced mild neurocognitive disorder.

Other (or unknown) substance-induced obsessive–compulsive and related disorder.

Other (or unknown) substance-induced sexual dysfunction.

Other hallucinogen intoxication.

Other hallucinogen-induced anxiety disorder.

Phencyclidine intoxication.

Phencyclidine-induced anxiety disorder.

Sedative, hypnotic, or anxiolytic intoxication.

Sedative-, hypnotic-, or anxiolytic-induced anxiety disorder.

Sedative-, hypnotic-, or anxiolytic-induced mild neurocognitive disorder.

Sedative-, hypnotic-, or anxiolytic-induced sexual dysfunction.

Amphetamine (or other stimulant) induced anxiety disorder.

Amphetamine (or other stimulant) induced obsessive–compulsive and related disorder.

Amphetamine (or other stimulant) induced sexual dysfunction.

Amphetamine or other stimulant intoxication.

Caffeine-induced anxiety disorder.

Cannabis intoxication.

Cannabis-induced anxiety disorder.

Cocaine intoxication.

Cocaine-induced anxiety disorder.

Cocaine-induced obsessive–compulsive and related disorder.

Cocaine-induced sexual dysfunction.

Hallucinogen persisting perception disorder.

Inhalant intoxication.

Amphetamine (or other stimulant) induced sleep disorder.

Caffeine-induced sleep disorder.

Cocaine-induced sleep disorder.

Inhalant-induced anxiety disorder.

Opioid-induced sleep disorder.

Sedative-, hypnotic-, or anxiolytic-induced sleep disorder.

Other (or unknown) substance-induced sleep disorder.

292.90 Amphetamine (or other stimulant) induced psychotic disorder.

Cannabis-induced psychotic disorder.

Cocaine-induced psychotic disorder.

Inhalant-induced psychotic disorder.

Other (or unknown) substance-induced psychotic disorder.

Other hallucinogen-induced psychotic disorder.

Phencyclidine-induced psychotic disorder.

Sedative-, hypnotic-, or anxiolytic-induced psychotic disorder.

Unspecified caffeine-related disorder.

Unspecified cannabis-related disorder.

Unspecified hallucinogen-related disorder.

Unspecified inhalant-related disorder.

Unspecified opioid-related disorder.

Unspecified other (or unknown) substance-related disorder.

Unspecified phencyclidine-related disorder.

Unspecified sedative-, hypnotic-, or anxiolytic-related disorder.

Unspecified stimulant-related disorder.

Unspecified tobacco-related disorder.

Amphetamine-related disorder NOS.

Nicotine-related disorder NOS.

### Unspecified mental disorder (n = 58)

300.90 Unspecified mental disorder.

Other specified mental disorder.

Mental disorder NOS.

The merging of DSM-IV and DSM-V diagnoses resulted in 31 categories of mental disorders used in the network analysis of mental disorders. Due to exclusion of certain diagnostic categories, 68 patients no longer belonged to any of the categories included in the analysis. Data of 5,065 patients were included in the network analysis.
